# Exploring the quiet eye in archery using field- and laboratory-based tasks

**DOI:** 10.1007/s00221-017-4988-2

**Published:** 2017-06-28

**Authors:** Claudia C. Gonzalez, Joe Causer, Michael J. Grey, Glyn W. Humphreys, R. Chris Miall, A. Mark Williams

**Affiliations:** 1 Centre for Cognitive Neuroscience, College of Health and Life Sciences, Brunel University London, Uxbridge, Middlesex UB8 3PH UK; 2 Research Institute for Sport and Exercise Sciences, Liverpool John Moores University, Tom Reilly Building, Byrom Street, Liverpool, Merseyside L3 3AF UK; 3 School of Sport, Exercise and Rehabilitation Sciences, University of Birmingham, Edgbaston, Birmingham, B15 2TT UK; 4 Department of Experimental Psychology, Tinbergen Building, 9 South Parks Road, Oxford, OX1 3UD UK; 5 School of Psychology, University of Birmingham, Edgbaston, Birmingham, B15 2TT UK; 6 Department of Health, Kinesiology, and Recreation, College of Health, The University of Utah, Salt Lake City, UT 84112 USA

**Keywords:** Gaze, Experts, Aiming, Programming, Attention

## Abstract

The ‘quiet eye’ (QE)—a period of extended gaze fixation on a target—has been reported in many tasks that require accurate aiming. Longer quiet eye durations (QEDs) are reported in experts compared to non-experts and on successful versus less successful trials. The QE has been extensively studied in the field; however, the cognitive mechanisms underlying the QE are not yet fully understood. We investigated the QEDs of ten expert and ten novice archers in the field and in the laboratory using a computer-based archery task. The computer task consisted of shooting archery targets using a joystick. Random ‘noise’ (visual motion perturbation) was introduced at high and low levels to allow for the controlled examination of the effects of task complexity and processing demands. In this computer task, we also tested an additional group of ten non-archers as controls. In both field and computer tasks, eye movements were measured using electro-oculography. The expert archers exhibited longer QED compared to the novice archers in the field task. In the computer task, the archers again exhibited longer QEDs and were more accurate compared to non-archers. Furthermore, expert archers showed earlier QE onsets and longer QEDs during high noise conditions compared to the novices and non-archers. Our findings show skill-based effects on QED in field conditions and in a novel computer-based archery task, in which online (visual) perturbations modulated experts’ QEDs. These longer QEDs in experts may be used for more efficient programming in which accurate predictions are facilitated by attention control.

## Introduction

Scientists examining the gaze behaviours employed by expert performers across several domains have improved our understanding of the perceptual–cognitive mechanisms that are characteristic of skilled performance (for reviews, see Mann et al. 2007; Rienhoff et al. 2016). In the field of sports science, for example, researchers have shown that, in certain sports, expert performers often employ fewer fixations of longer durations compared with non-experts, resulting in a more efficient extraction of task-relevant information (Mann et al. 2007; Williams and Davids 1998; Williams et al. 1994). Similarly, Vickers (1992) highlighted distinct gaze patterns between expert and novice golfers while performing putts and identified that experts kept a steady gaze or “quiet eye” (QE) at a specific location before ball contact. The QE was subsequently formally defined as the duration of the final fixation or tracking gaze on a target within a threshold of 3° (or less) and has a minimum duration of 100 ms. The onset of the QE occurs prior to the final action of the task and the offset is identified when eye movements fall outside the threshold (Vickers 1996).

Furthermore, with the use of video-based mobile eye trackers, longer quiet eye durations (QEDs) have been reported to be characteristic of experts compared with non-experts, and on successful compared with less successful performance, in many aiming sports, including shooting (Causer et al. 2010), darts (Rienhoff et al. 2013), and billiards (Williams et al. 2002). In addition, the QE has been successfully used as a training tool (where to look and for how long) with improvements in performance linked to relative increases in QED (see Vine et al. 2014) in different targeting sports (Causer et al. 2011; Moore et al. 2012; Vine and Wilson 2011) and, recently, both in the training of surgical skills (Causer et al. 2014) and motor skills in clinical populations (Miles et al. 2015).

A number of mechanisms have been proposed to explain the QE and its effect on performance. Published reports support a programming hypothesis (Horn et al. 2012; Mann et al. 2011; Williams et al. 2002). In line with this hypothesis, the QE period is suggested to facilitate information processing and its duration is thought to reflect the time needed to programme and fine-tune a movement response. Thus, longer QEDs are thought to extend this critical motor preparation period, enhancing performance (Mann et al. 2011; Vickers 2011). Williams et al. (2002) reported longer QEDs with increased levels of task complexity, when manipulating the distance of a billiards shot (near versus far) and the time allowed to complete a specific shot (constrained versus unconstrained time). Their findings support the programming hypothesis, in that longer QEDs correspond to the greater information processing demands for complex tasks, requiring longer programming times. However, this very general explanation does not fully describe the positive facilitatory effects of the QE or define the actual information that is being processed (Gonzalez et al. 2015). Furthermore, the notion that experts have longer QEDs reflecting prolonged attention and motor preparation time questions whether only open-loop programming mechanisms are active during this extended time (Vine et al. 2013).

Current models of motor control suggest that skilled behaviour relies on a combination of sensory feedback and predictions of both our own body and the tools we interact with to accurately estimate the consequences of a motor response (Wolpert and Flanagan 2001). The combination of the two streams of information (motor prediction and sensory feedback) enhances perceptual–motor performance (Shadmehr et al. 2010), since making inaccurate predictions or solely relying on feedback can be costly in terms of accuracy and timing. In line with this model, researchers examining QE mechanisms and their effects on performance have suggested that longer QEDs facilitate programming and that the inclusion of online control mechanisms under visual guidance aids the maintenance of the QE (Causer et al. 2016; Vine et al. 2013, 2017). This latter conclusion is in accordance with QED findings which suggest that late information pickup is important for accuracy, and continued gaze control is critical for preventing performance failure in expert golfers (Vine et al. 2013). Causer et al. (2016) examined the effects of online control using visual occlusion during movement initiation in a golf putting task and found that performance on a putting task suffered without the availability of visual online control during the QE. They concluded that the QE reflects programming and the inclusion of online control, but that online control is critical to performance. Furthermore, having continuous online information available to make predictions has been shown to benefit performance overall, since the accuracy of the internal representations (e.g. of the target) may decay over time (Heath and Binsted, 2007).

Klostermann et al. (2013) investigated the performance-enhancing effects of experimentally manipulated QEDs in an externally paced throwing task by presenting a target at different timings (short and long presentations, similar to Williams et al. 2002) and locations (random and predictive) during movement unfolding. The facilitatory effects of longer QEDs were apparent only under a high information processing load (short and random target presentations) and that QED effects on performance seemed to disappear with increased predictability of the target’s location and decreased task demands. Klostermann et al. (2013) argued that the predictability of the target might have facilitated relevant information processing (as early programming) that was not required during the QE period; consequently, long QEDs were “dispensable” under conditions of high predictability and low task demands, compared to when the target was perturbed into random locations. Moreover, in the less demanding task, the lack of QED and performance differences between the short and long target presentations reflected the availability of crucial online movement control for the responses. However, they suggested a need for further QE studies that focus on disturbing these online mechanisms. In line with Williams et al. (2002) and Horn et al. (2012), Klostermann et al. described their findings as part of the information processing explanation, but noted that the exact nature of these processing demands was not known and results could be explained by attention control mechanisms (i.e. in random target presentations, attentional costs for late stimulus identification were high). Thus, programming alone may not result in performance-enhancing QEDs.

In regards to attention control, Vickers (2009) suggested that the QE involves top-down (dorsal stream) control mechanisms to guide attention and programme a response, while suppressing intrusive bottom-up (ventral stream) responses. In line with this inhibition hypothesis, Klostermann et al. (2014) examined the links between attention and QED and suggested the involvement of additional functions during this steady gaze period, mainly, (inhibitory) attention control mechanisms to explain the facilitatory effects associated with a longer QED. The inclusion of inhibitory mechanisms is in accordance with the control and maintenance of attention and reflects higher-order cognitive control (Deubel and Schneider 1996; Findlay 2009; Rizzolatti et al. 1987), which may be implemented during the QE (Gonzalez et al. 2015). In addition, attention control strategies are those that have mainly been implemented in QE training studies, resulting in improvements in performance (see Vine et al. 2014). Inhibitory control may allow experts to select the most relevant information, maximizing the speed and accuracy of online processing to better predict a motor response (motor programme). Thus, experts rely on predictions to filter sensory information (Wolpert and Flanagan 2001) and to produce timely responses rather than solely relying on sensory input, which is slower and more susceptible to noise.

Given that QED effects have been associated with complex tasks that require online control and late information pickup, it may be that continuous monitoring of (afferent) signals, including visual and proprioceptive information, is integrated during the QED and afforded by gaze and attention control mechanisms. The difficulty in studying such behaviour lies in the fact that field manipulations to examine the proposed underlying mechanisms are limited. Furthermore, establishing a relationship between QED and performance may be difficult in the field due to the fact that differences in performance accuracy may not necessarily be directly related to a specific QED or to the high variability that may be encountered in this environment, such as the existence of background distractions and/or distinct differences in movement characteristics when shooting or throwing between groups. However, since QED effects have been observed under more controlled conditions (e.g. Klostermann et al. 2013), it seems promising that the mechanisms underlying the QE can be explored in a carefully controlled laboratory environment, away from the sports field. For example, Behan and Wilson (2008) implemented a computer archery task completed by non-experts under two anxiety conditions. Their results replicated previous in situ findings related to the effects of anxiety on the QED and pointed to links between attention control and QE, although these effects (as well as Klosterman et al’s 2013 findings) were not related to expertise per se. Given that the QE has been described within the expertise model, controlled investigations into this gaze strategy in experts compared to non-experts may provide more accurate knowledge of the QE.

In this paper, we had two aims. First, we examined skill-related differences in QED between expert, Olympic-level archers and novice archers in the field. To our knowledge, this is the first attempt to examine distinct aiming gaze behaviour between experts and novices in the sport of archery. To achieve this aim, we measured gaze (QED) and performance (radial error and scores). We expected skill-related QED differences in accordance with previous research in aiming sports, with experts having a longer QED and better accuracy (e.g. Causer et al. 2010; Rienhoff et al. 2013).

Our second aim was to design a computer-based archery task that would allow for a controlled examination of QED and performance across our expert and novice archers and would replicate the QED field results. Moreover, we aimed to minimize field ‘noise’ variability (e.g. differences in technique, location, equipment), isolate expert archers’ gaze aiming strategies (from motor expertise), and examine how these would impact performance. In line with the current theories of online integration and attention control, we manipulated task difficulty in the computer-based task using two conditions involving two different levels of visual perturbation while participants attempted to aim at an archery target. More specifically, we implemented a ‘high noise’ (HN) condition in which the requirement of online motor control was higher than in a less difficult and more predictable ‘low noise’ (LN) condition by perturbing the aiming crosshair. Unlike Behan and Wilson’s (2008) simulated experiment which included an anxiety manipulation and non-archers, we included archery experts, novices, and an additional control group (non-expert, non-archer) to better identify expert-related QED differences in our computer task. We hypothesised similar results to those expected in the field in that the expert archers would show a longer QED than novice archers and non-archers indicating that some of the gaze strategies used by experts can be identified and examined in a computer task. Also, we predicted longer QEDs in the more complex HN condition when compared with the LN condition. We further hyopthesized that differences in QEDs across groups would reflect the ability to override the added crosshair’s noise (visual perturbation) and continuous updating, which could lead to temporal lags and errors in performance, particularly in the HN condition.

## Methods

### Participants

We recruited 30 participants. The expert group comprised ten Olympic-level archers from the Team GB Archery team who had at least 2 years of competitive experience at international level (mean age and SD: 26 ± 10.02 years; seven males and three females; mean archery experience: 11.42 ± 5.97 years; mean training time: 34.45 ± 7.52 h/week). The ten novice archers were members of the university archery club who had experience of at least one national inter-university competition (age: 31.09 ± 13.56 years; six males and four females; mean archery experience: 1.98 ± 0.94 years and a mean training time: 4.36 ± 2.83 h/week). The ten non-archers were university students (age: 25.5 ± 2.51 years; six males and four females), recruited from the undergraduate population. All participants were right-handed, had normal eyesight and no known neurological or developmental conditions. None of the archers reported any experience in computer archery or any other aiming computer “games” and only two non-archers reported using video games regularly (<4 h/week). This study was approved by the lead university’s local ethics committee and conducted in accordance with the ethical standards laid out in the 1964 Declaration of Helsinki. All participants provided informed consent.

### Procedure

There were two experimental sessions involving a field and a computer task, respectively. For the field task, expert and novice archers were asked to shoot 24 arrows at an 80 cm archery target (ten multi-colour rings) located at 30 m distance (corresponding to 1.5° of visual angle for the target’s diameter). The individual session commenced with a warm up (12 arrows at 10 m distance), followed by 12 practice shots at the 30 m target. For the experimental trials, archers were asked to shoot six arrows in a row, at will, in four blocks (24 arrows in total). Participants were able to see in which target ring their arrows landed (feedback) and were aware that their accuracy was recoded. The typical archery target has ten rings and each ring corresponds to a score ranging from 10 to 0, with 10 being the highest score corresponding to an arrow shot on target centre or “bull’s-eye”, a score of 1 for the last outer ring, and a score of 0 for a complete miss. Rest breaks were provided in between each block, at which time the arrows were collected. The session lasted for approximately 90 min.

The computer task was designed using a custom made programme (psychtoolbox 3, Matlab 2013a, The Mathworks, Inc). For this task, archers and non-archers were asked to shoot at an archery target (10 multi-colour rings, 80 pixels in diameter) presented on a computer laptop (900 × 1600, 60 Hz, 15″ Samsung Electronics Co), using a joystick (two axis, Sidewinder, Microsoft Co). Participants were seated on an adjustable chair 60 cm from the computer screen (for an equivalent 1.5° of visual angle for the target’s diameter, thus each ring was eight pixels or 0.15˚ of visual angle) and rested their head on a chinrest to avoid head movements. Participants held the joystick, which was located in front of them, with their right hand. The joystick could be moved along the *Y* axis (upwards direction by pulling the joystick and downwards by pushing) and the *X* axis (right and left).

**Fig. 1:**
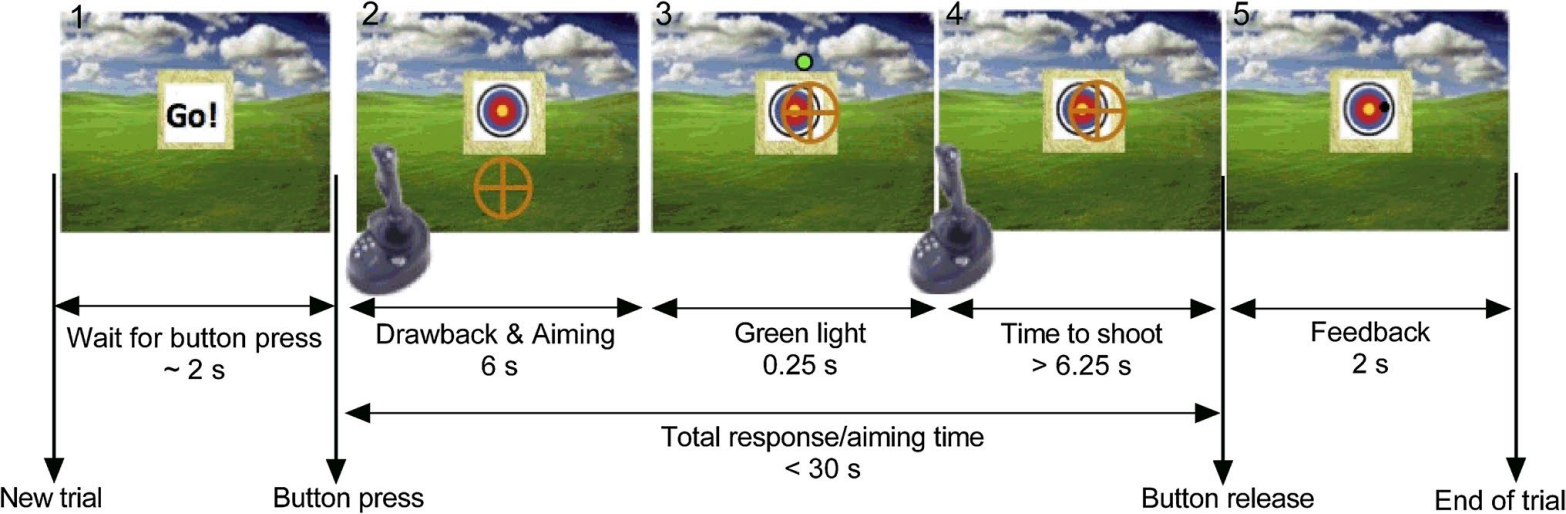
The image shows the computer experiment with crosshair (representing joystick position), the target (made of ten multi-coloured circles, equivalent to 1.5° of visual angle, thus, 0.15° per ring) presented on the centre of the screen. The first screen image (*1*, *left*) shows the “Go!” signal which alerted the participant of a new trial that commenced once the participant pressed the joystick’s front button and corresponded to the start of the aiming time. The second screen image (*2*, *left*) corresponds to when the participants pressed the joystick’s front button, which made the crosshairs appear at the lower part of the screen (4° from the target centre). At this time, participants had to pull the joystick to elevate it towards the target (drawback) and continue to aim at the target, while waiting for the green light to appear. The third image shows when the green light appeared after the 6 s of this continued aiming and flashed for a total duration of 0.25 s. The fourth image shows the time given to participants to shoot (button release) at the target after the green light had disappeared (6.25 s within the 30 s limit). The fifth and final image (*5*, *right*) shows where the arrow landed on the target after button release as feedback (2 s). The sizes of ten ring target (two rings per colour), background, green cue, and crosshair shown are for schematic purposes and are not to scale

The experiment commenced with a visual ‘Go!’ signal, which indicated that the participant had to press the joystick’s front button with his or her index finger, at which time, the target and crosshair (100 pixels in diameter) appeared, corresponding to the start of aiming time (Fig. 1). Participants then raised the crosshair to aim at the target by pulling the joystick backwards, while maintaining the joystick’s front button pressed, to simulate drawback and elevation. Once they positioned the crosshair on the target, they were instructed to continue aiming at the target until a green ‘light’ flashed briefly above the target, 6 s after movement onset and for 250 ms. The green light indicated that participants were allowed to shoot at the target by releasing the button after the light disappeared (6.25 s after button press). Participants were instructed not to look at the green light directly and continue aiming, but to be aware of it by using peripheral vision. The green light set a minimum aiming time, which was implemented to avoid a quick ‘pass-by’ shooting (i.e. moving the crosshair across the target and guessing when to best release it) and forced the participants to actively aim at the centre of the target, as they would in the field. Participants became habituated to this constraint after only a few trials. They were also made aware that they had a 30 s time limit to shoot in total, from when they started aiming. However, none of the participants reached this limit. After the button was released, a simulated arrow followed a parabolic trajectory to the target that reflected any positional error at the moment of release, and participants were able to see where the ‘arrow’ had landed on the target (feedback) within 2 s.

Participants performed the computer task under two conditions with distinct task complexities, namely, high noise (HN) and low noise (LN). This visual manipulation consisted of inducing random movement or ‘noise’ into the crosshair’s path as the participants attempted to aim at the target by moving the joystick. A set of 30 random *X* and *Y* paths were generated with a random number generator and two different low pass filters were applied to create either a smoother, more predictable or a jerkier, less predictable 2D motion applied to the crosshair’s path. To make sure that the participants were familiar with the joystick and task, a total of 20 practice trials (10 high noise, 10 low noise) were given prior to experimental trials. There were a total of 60 experimental trials in which the HN and LN conditions were alternated every 15th shot (counterbalanced). Participants were told to aim and shoot at the target’s centre or “bull’s-eye” after the green light disappeared but within the 30 s limit. For motivation, we alerted participants to the fact that their accuracy would be recorded. The computer task session lasted for approximately 60 min.

### Measures

#### Gaze

Eye movements were measured using an electro-oculogram (EOG) bio-amplifier (gain of 100) with a band pass range from DC to 500 Hz (ADInstruments Ltd, Oxford, UK). The analogue data were sampled at 1000 Hz and recorded online using PowerLab data acquisition system and LabChart 5 software (ADInstruments Ltd, Oxford, UK). We implemented EOG techniques since the small electrodes placed on the archers’ face did not interfere with their aiming and due to the fact that some archers tended to close an eye or squint when aiming, leading to data loss when pilot testing using a video-based, mobile eye tracker. In addition, head movements in archery are minimal. Thus, EOG measures were better suited for our experiments, providing information on eye movement amplitude measured relative to target centre as well as temporal information important for QE (i.e. fixation duration, QE onset and offset).

To measure horizontal and vertical eye movements (EOGh and EOGv), two Ag/Ag Cl electrodes were placed over the participant’s right and left temples and two electrodes were placed above (on forehead) and below the right eye. A ground electrode was placed over the back of the left ear. Pre-processing of EOG signals were performed off-line using LabChart 5 (ADInstruments Ltd) and further analyses to identify QE were performed using custom-made programmes (for field and computer tasks) in Matlab (The Mathworks, Inc). EOG signals were bandpass filtered with low and high cutoff frequencies of 0.2–30 Hz, respectively, to de-noise and remove drift from the signal (Marmor et al. 2011). Blinks were eliminated from each trial and the gaps were linked using linear interpolation.

Calibrations were performed in the field after each experimental block using the same target stand at 30 m. Participants fixated at a marked centre target (3 cm in diameter and visible to participants) for 2 s and then moved their eyes to fixate at marked right, left, upper and lower targets for 2 s, while returning to the centre target between each location (1° movements for each location). This process was repeated twice for consistency for each block. For the computer task, following a centre fixation (2 s), 16 horizontal (8 right, 8 left) and 16 vertical (8 upwards and 8 downwards) cross targets (0.5 × 0.5 cm or 50 × 50 pixels) were presented sequentially for 2 s each, with separation of 0.5˚ and the participant returning to the centre fixation point between each target.

The horizontal and vertical voltages at each positional jump in these calibrations (with respect to centre and averaged over 1 s at each location) were recorded for each participant. Linear regressions were performed on the EOG data (see Berret et al. 2014) and the resulting slopes (averaged for EOGv and for EOGh) were used to calculate the voltages that were equivalent to 1° for each participant. These EOGv and EOGh voltages were used as eye movement thresholds to identify the QE timings.

#### Quiet eye

We defined the QED as the period in which eye movements were maintained within 1° of visual angle (0.5 and −0.5˚ of visual angle relative to target centre) and for at least 100 ms prior to arrow release. Our QE definition did not include larger saccades that were outside of the target area also keeping within the definition of the QE as a fixation (see Gonzalez et al. 2015).

*Field task*- In the field tests, electromyography (EMG) techniques were used to measure muscle activation and identify the time at which the arrow was released. Ag/Ag Cl electrodes were placed over the forearm, on the flexor digitorium superficialis (FDS) muscle with a ground electrode over the elbow. This signal was bandpass filtered with cutoff frequencies of 10–500 Hz, collected into the PowerLab system (also sampling at 1000 Hz, using a bio-amplifier, gain 100) and recorded with LabChart 5 software (ADInstruments Ltd, Oxford, UK).

To identify the trial epochs, the EMG signal was rectified and low pass filtered with a cutoff frequency of 10 Hz to obtain the linear envelope. The start of the trial was identified as the time at which 50% of maximum FDS activation was achieved as a result of the draw back movement to start aiming. The sudden decrease in FDS muscle activation as a result of releasing the arrow was identified using the peak of the derivative of the EMG linear envelope. The start of aiming was always set to 0 s and the absolute difference between this start time and arrow release time was defined as the total response time (TRT). Eye movements were inspected during TRT derived from the EMG signal in Matlab. The QE onset and offset were identified by working backwards from arrow release time until either the vertical or horizontal EOG signal fell outside the 1° boundaries (0.5 and −0.5° relative to target centre).

**Fig. 2:**
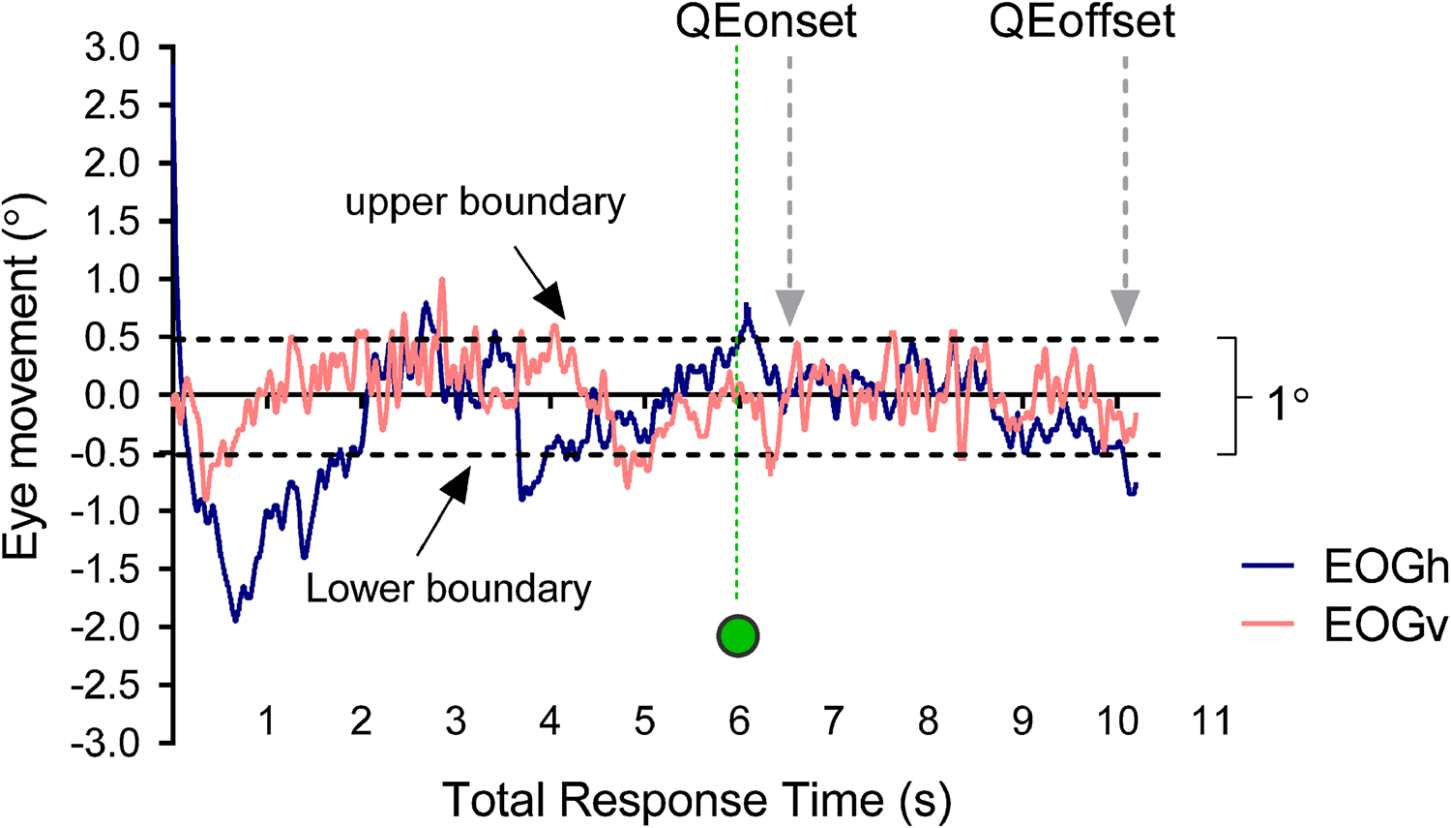
An example of a participant’s eye movements corresponding to vertical (EOGv) and horizontal (EOGh) traces (converted to ° of visual angle from EOGh and EOGv voltage calibration regressions) during the total response time (TRT). The QE boundaries are set at a 1° threshold (0.5 and −0.5 from target centre). Eye movements fall outside of the 1° boundary, thus establishing a QE onset/offset. The QE onset was identified after the green light which appeared after 6 s of aiming and for a duration of 250 ms. Blinks were eliminated and typically spotted outside the QE

*Computer task*- For the computer task, the joystick’s button press was defined as the start time and button release corresponded to the arrow release. These event signals were fed into the data acquisition box and used as digital markers together with the EOG signals. As with the field task, the TRT was defined as the difference between arrow release and start time. Eye movements were inspected during this TRT, and the QE onset/offset timings were identified by working backwards from arrow release time. In addition, the resulting EOGv and EOGh voltages obtained from the calibration were used to identify the corresponding value for 1° (0.5 and −0.5° from target centre) for QED onset and offset detection (Fig. 2). Trials in which there was no QE or the QED was less than 100 ms were excluded from all analyses. We additionally calculated QED as a percentage of TRT (QED%) for the field and the computer task to investigate the amount of time that each group exhibited a QE relative to the total aiming time prior to arrow release. These QED% values were calculated to make sure that aiming time did not account for group differences.

#### Performance

The main performance measure was obtained by measuring the radial error (RE in ° of visual angle) from each arrow’s *X* and *Y* location to the target centre. The corresponding archery scores were also measured as a 10–0 scale based on the final location (ring colour) in which the (real and simulated) arrows landed (e.g. score of 10 for centre, 1 for outer ring, and 0 for a miss).

*Field task*- An 80 cm blank sheet of paper was placed behind the target sheet to record arrow penetrations. New blank sheets were placed after each block of six arrows for each participant. In addition, notes of the location of the arrow were taken after each shot, using binoculars to identify arrow number/location. The *X* and *Y* locations of the 24 arrows for each participant were measured by hand using a graph ruled sheet. The REs from target centre were calculated and converted to degrees of visual angle by multiplying each value by 0.019°, which corresponded to 1 cm.

*Computer task*- The joystick’s *X* and *Y* positions at the time of button release were recorded automatically by the Matlab program and the RE from the target’s centre was subsequently calculated. Accuracy measures were converted to degrees of visual angle by multiplying each value by 0.019°, which corresponded to one pixel. In addition, participants’ scores (also raging from 10 to 0) were computed based on what colour ring the arrow landed on (eight pixels or 0.15° per ring).

Trials in which participants shot prior to the green light (<6 s of aiming) were eliminated and these early responses corresponded to 3.80 ± 1.13% of all experimental trials in experts, 3.75 ± 1.17% in novices, and 4.0 ± 1.25% in non-archers.

### Statistical analyses

For the field task, separate between-participants ANOVAs were used to analyse the overall QED and performance (RE). In addition, *t* tests were implemented to compare QE onset/offset timings in the field between expert and novice archers. A second set of between-groups repeated measures ANOVAs were used to analyse QE onset/offset, overall QED, and trial accuracy (RE) across the HN and LN conditions in the computer task. The between-participant factor in our analyses was experts and novices for the field task and expert archers, novice archers, and non-archers for the computer task. The QED%, TRT, and performance scores are presented together with field results in Table 1. Pearson correlations were implemented to determine expert-related QED and performance relationships. Significant effects and interactions were evaluated using Bonferroni corrected post hoc tests. A significance level of *α* = 0.05 was established for all statistical analyses. The results and graphs are expressed as means ± standard errors of the mean (SE). Effect sizes are reported as partial eta-squared values and Cohen’s *d* values when appropriate. Sample sizes were computed via a priori power calculation based on pilot data and a sample size of six participants for each group was required for a *β* = 0.8, *α* = 0.05 and an interaction with an effect size of 1.0.

**Table 1:** A summary of the results for field and computer tasks

Group	QED (s)			QED %			RE (°)			Score			TRT (s)		
	Field	LN	HN	Field	LN	HN	Field	LN	HN	Field	LN	HN	Field	LN	HN
Expert															
Mean	1.9	2.6	3.3	29.4	17.3	18.2	0.10	0.20	0.30	9.1	8.0	6.7	6.3	16.0	20.0
±SE	0.3	0.2	0.4	2.9	1.4	1.7	0.04	0.02	0.01	0.3	0.3	0.4	0.7	1.5	6.6
Novice															
Mean	0.7	2.3	2.0	18.1	18.7	15.2	0.30	0.20	0.30	6.8	6.4	7.7	4.4	14.0	14.0
±SE	0.1	0.3	0.2	2.6	1.4	1.7	0.04	0.09	0.02	0.6	0.2	0.2	0.3	3.4	1.1
Non-Archer															
Mean	–	1.2	1.2	–	9.4	10.1	–	0.3	0.5	–	6.6	5.3	–	12.0	13.0
±SE	–	0.1	0.1	–	1.8	0.6	–	0.02	0.03	–	0.3	0.3	–	0.6	1.2

*QED* quiet eye duration, *QED%* quiet eye duration as a percentage of trial duration or aiming time, *RE* radial error, *mean Score* 0–10 range based on ring location, *TRT* total response time, *LN* low noise, *HN* high noise conditions

## Results

### Field task

**Fig. 3:**
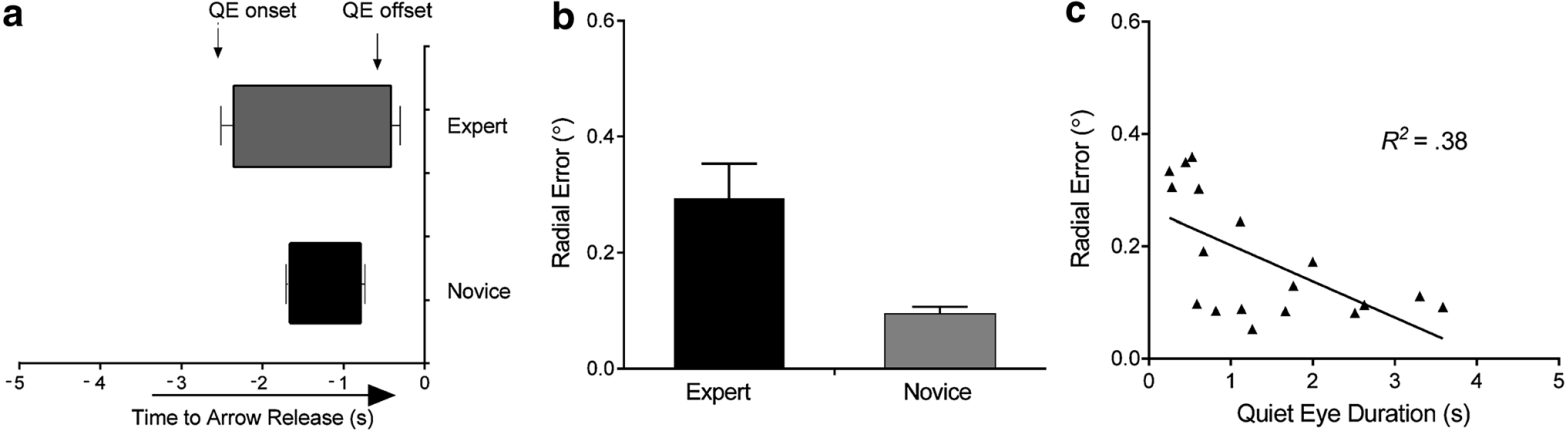
Field task quiet eye onset and offset (**a**) across aiming time, radial error (**b**) between experts and novices and quiet eye duration (QED) and performance (radial error) correlations across all archer groups (**c**). *Graph* **a** depicts QE onset/offset timings which were examined by moving backwards from arrow release. QE timing analysis **a** showed earlier onsets and, thus, longer QEDs in experts compared to novices, *p* < 0.5. Experts were more accurate and, in addition, a significant correlation **c** showed that longer QEDs corresponded to better performance, *p* < 0.5

An analysis of QE timings (onset and offset) revealed that experts had earlier QE onsets compared to novices, *t*(20) = 3.66, *p* = 0.002, *d* = 0.86. The QE offsets were not significantly different (*p* = 0.057; Fig. 3a). This early onset indicated that experts had overall longer QED, *F*(1,20) = 8.09, *p* = 0.012, *η*_p_^2^ = 0.35, compared to novice archers (1.96 ± 0.34 and 0.76 ± 0.1 s, respectively, see Table 1). In addition, it was revealed that experts were more accurate, *F*(1,20) = 18.42, *p* = 0.001, *η*_p_^2^ = 0.55, compared to novice archers (Fig. 3b).

A Pearson correlation between QED and performance (RE) across all participants revealed a significant linear relationship, *R* ^2^ = 0.38, *p* = 0.006, which showed that longer QEDs corresponded to smaller errors (Fig. 3c).

### Computer task

**Fig. 4:**
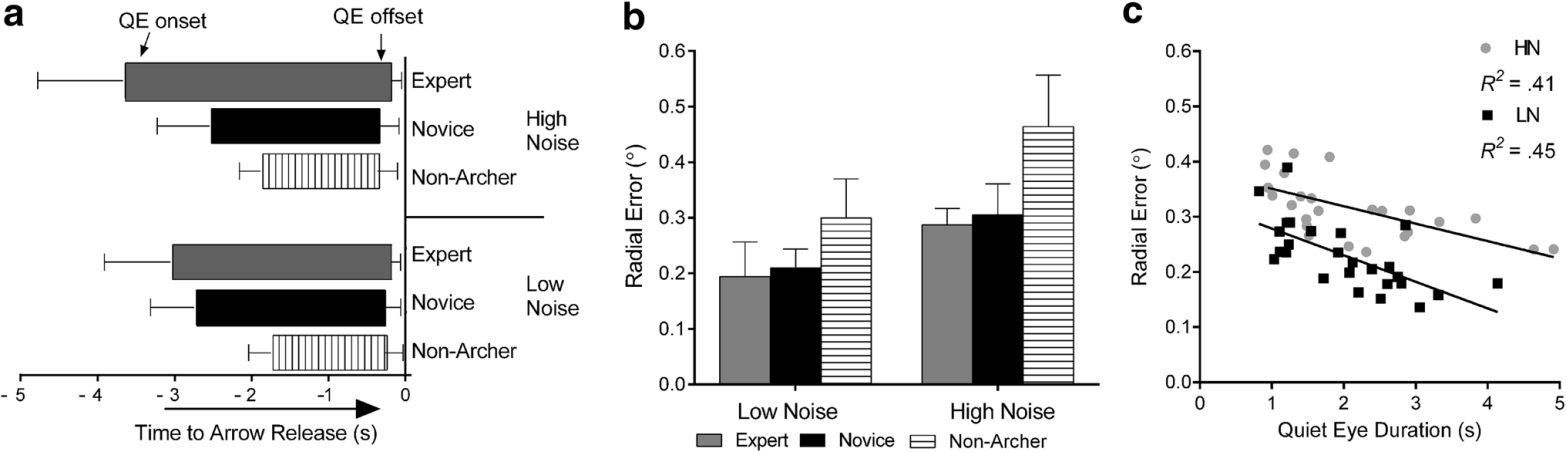
Computer task QE onset and offset (**a**), radial errors (**b**) between experts, novices, and non-archers across high noise (HN) and low noise (LN) conditions; and correlations between QED and performance (**c**) across all groups. Experts again showed earlier QE onsets and longer QEDs in the HN, while non-archers showed the latest onsets and shorter QEDs in the LN (**a**), *p* < 0.5. Radial errors showed that all groups performed better in the LN compared to the HN and that archer groups exhibited lower errors compared to non-archers, *p* < 0.5 (**b**). As in the field task, significant correlations were observed in the LN and the HN tasks, *p* < 0.5 (**c**)

An analysis of QE timings (onset and offset) revealed significant differences in the QE onsets, but not in the offsets. There was a significant group × noise condition interaction for QE onsets, *F*(2, 27) = 9.73, *p* = 0.001, *η*_p_^2^ = 0.44. The expert archers showed earlier QE onsets in their HN compared to their LN condition, and had earlier onsets compared to novices, *p* = 0.006, and non-archers, *p* < 0.001, in the HN condition (Fig. 4a). In addition, non-archers showed the latest onsets of the group in the LN, *p* < 0.001 and *p* = 0.007, compared to novices and experts, respectively.

In regards to the absolute QED, the early onsets meant that expert archers showed significant increases in their QEDs, *F*(1,27) = 11.96, *p* < 0.001, *η*_p_^2^ = 0.49, between LN (2.6 ± 1.6 s) and HN (3.3 ± 0.41 s) conditions, *p* = 0.001. The expert archers’ early onsets also resulted in longer QEDs compared to novices in the HN condition (1.9 ± 0.21 s), *p* = 0.002. In addition, non-archers had significantly shorter QED compared to experts, *p* < 0.001, and novices, *p* = 0.001, in the LN (1.19 ± 0.5 s) condition and shorter from experts in the HN (1.2 ± 0.1 s) condition, *p* < 0.001, but not different when compared with the novices in this HN condition, *p* > 0.05.

Performance measures (RE) revealed a main effect for noise condition, *F*(1,27) = 34.48, *p* < 0.001, *η*_p_^2^ = 0.58, and group, *F*(1,27) = 6.91, *p* = 0.004, *η*_p_^2^ = 0.36. Participants were more accurate in the LN compared to the HN condition (Fig. 4b). In addition, archers were more accurate compared to non-archers, *p* = 0.009 and *p* = 0.015, for expert and novice archers versus non-archers, respectively. However, there were no RE differences between the expert and novice archer groups, *p* > 0.999.

Significant correlations were observed between QED and RE in both HN, *R* ^2^ = 0.41, *p* < 0.001, and LN, *R* ^2^ = 0.45, *p* < 0.001, conditions across expert, novice, and non-archer groups (Fig. 4c). Thus, longer QEDs corresponded to enhanced accuracy in both noise conditions.

### Field and computer task

The results (mean ± SE) across both field and computer tasks are presented in Table 1. An analysis of QED% on the field task replicated absolute QED results in which experts showed longer QED relative to trial duration, *F*(1,20) = 6.8, *p* = 0.02, *η*_p_^2^ = 0.31. There were no differences in trial duration between the two archer groups, *p* = 0.153. In addition, experts had higher scores, *F*(1,20) = 12.38, *p* = 0.003, *η*_p_^2^ = 0.45, compared to novice archers.

The results from the computer task revealed a significant noise condition by group interaction in QED%, *F*(1,27) = 7.11, *p* = 0.004, *η*_p_^2^ = 0.37. Post hoc tests showed significant group QED% differences in the LN (non-archers versus experts and novice archers, both *p* < 0.001) and in HN, *p* = 0.002, conditions. Thus, archers (experts and novices) had a longer QEDs relative to the trial duration compared to non-archers. Our analysis also revealed that computer task scores were higher, *F*(1,27) = 111.08, *p* < 0.001, *η*_p_^2^ = 0.81, and TRTs were longer, *F*(1,27) = 7.81, *p* = 0.01, *η*_p_^2^ = 0.23, in the HN compared to the LN condition. As with RE measures, a group effect, *F*(1,27) = 10.77, *p* < 0.001, *η*_p_^2^ = 0.46, showed that experts and novice archers had better scores compared to non-archers, *p* = 0.001 and *p* = 0.003.

## Discussion

We report a novel attempt to examine the QED in archery, both in the field setting and using a computer-based archery task. We predicted, based on previous research, that the expert archers would show longer QED than novice archers (e.g. see Causer et al. 2010; Williams et al. 2002). Our findings from the archery field task are in accordance with these previous reports indicating longer QEDs in experts compared to novice archers during aiming tasks. In addition, correlation analysis across the archer groups indicated that longer QEDs corresponded to smaller errors. To our knowledge, this is the first report of QEDs in the sport of archery.

We expected that field results would be replicated in our computer task and that the addition of a non-archer group would allow us to make comparisons with different expertise levels (archers vs. non-archers and expert vs. novice archers) that could not be implemented in the field and would better indicate skill-related gaze strategies that result in superior performance. It should be noted that our computer task and the implementation of a visual perturbation of the aiming crosshair results in an artificial situation; it was not our intent to directly mimic archery nor to suggest that computer tasks could replace in situ training. Our intention was to examine the gaze strategies during our computer task in an effort to control for the amount of online information that needed to be processed, without the (motor and environmental) variability encountered in the field and between groups. With this novel manipulation, we expected to see longer QEDs in the HN compared with LN condition, which would provide support for the importance of online control during the QED period. We were also able to show that the QED in experts is modulated by task requirements. More specifically, experts seemed to be sensitive to the noise levels and prolonged their QED during higher task complexity (i.e. HN), while non-experts maintained their performance throughout the conditions. We argue, in line with previous suggested QE mechanisms mentioned in the introduction (e.g. Causer et al. 2016; Vine et al. 2017) and in accordance with models of skill-related motor control, that this extended time for processing incorporates more efficient online control. Furthermore, in our task, the type and processing of such information is already known, through previous published reports investigating online control through similar visual perturbations (see Sarlegna and Mutha 2015).

In the computer task, the noise introduced into the crosshair’s path varied from slower, smoother, and more predictable profiles in the LN condition to faster, jerkier, and more random profiles in the HN condition. Thus, in both conditions, participants were required to control the joystick during aiming until the arrow was shot; however, in the HN condition, enhanced monitoring, and greater error corrections were crucial to maintain the crosshair on the desired location (i.e. “bull’s-eye”) (Weir et al. 1989). Performance measures were in accordance with this task complexity effect and participants were overall less accurate in this HN task compared to the LN task. We also found group effects in the computer task, with the archers performing better compared to the non-archers. Radial errors indicated that the non-archers had greater difficulty in positioning the crosshair on or close to the target centre at the time when they decided to shoot, resulting in lower scores. These timing and accuracy issues may be explained by examining gaze behaviour during the task.

Our analysis of the eye movements showed that archers exhibited longer QEDs compared to non-archers, in accordance with previous findings showing that QEDs can discriminate between performance and expertise levels (see Gonzalez et al. 2015). However, while these field and computer task results do not imply a causal relationship between QED and performance, they do suggest that some aspects of the aiming strategy used by the archer groups were also implemented in our computer task. It is also possible that these skill-related gaze strategies (QE) may have resulted in superior performance. We propose that a steady gaze fixation or QE aided the archers in keeping track of the joystick’s position with respect to the target and to better predict when the crosshair would be on the target’s centre or positioned as close to the centre as possible. In contrast, the non-archers may have been distracted by the crosshair’s motion and their moment-to-moment joystick adjustments (online error corrections), resulting in shorter QEDs and larger errors. This suggestion is in accordance with the notion that skilled behaviour incorporates both predictions and (online) feedback, while non-archers may have relied more on momentary sensory input as they attempted to correct their errors (Weir et al. 1989; Wolpert and Flanagan 2001; Wolpert et al. 1998).

The QE timings did not reveal significant differences in QED offsets between the groups or noise conditions. This finding may be due to the addition of noise in the joystick’s path and the need to control the joystick at all times. In addition, offset times did not differ between groups in the field, suggesting that late sensory information was as important in the field as in the computer experiment. This result is in accordance with Klostermann et al.’s (2013) findings, which showed no significant differences in QE offset on their throwing task despite having different levels of final target location uncertainty. Thus, it is noted that in their experiment, information relating to the target came at a late time and, despite this, participants adopted an early QE onset, resulting in longer QEDs. Klostermann et al. (2013) also reported facilitatory effects of longer QEDs on performance during high task demand conditions, in which target uncertainty (random target presentation) was incorporated into the motor programme (i.e. increased information processing), presumably during the QE.

Körding and Wolpert (2004) provided evidence for the optimal integration of both predictions and sensory feedback for state estimations and suggested that with enhanced uncertainly there is higher reliance on predictions about the target, which are then matched to the actual feedback. Additionally, current research findings suggest that uncertainty about the target is taken into account for online adjustments in which the time available and the cost of making corrections are important factors (see Sarlegna and Mutha 2015). Thus, uncertainty increases variability in performance, but enhances the requirement for prediction about the target. In our experiment, it was the experts who revealed earlier QE onsets and longer QEDs in both computer and field tasks and this was more evident in the HN than LN condition in the computer task. An early QE onset may extend the information processing period of a particularly complex task, in accordance with the previously stated programming hypothesis (Williams et al. 2002). However, we further suggest that the experts’ earlier QE onsets, which resulted in longer QEDs, were employed to better accommodate the uncertainty of the crosshair’s movements to allow for better predictions during the aiming period. The novice archers also showed longer QEDs compared to controls, indicating that some of this additional processing was taking place.

In our computer task, uncertainty from the noise implemented into the crosshair’s path increased the demands for online corrections and modulated QE onsets and QEDs in expert archers. Others have suggested that rapid online corrections caused by changes in target location are somewhat low-level, ‘automatic’ responses that occur prior to voluntary corrections (Cameron et al. 2009; Day and Lyon 2000; Diedrichsen et al. 2004), but that these automatic adjustments may be selectively suppressed with learning (Gritsenko and Kalaska 2010). Experts may be able to suppress non-functional or time-consuming corrections early and implement more efficient volitional and predictive online control. Thus, archers implemented better predictions that resulted in lower error, facilitated by longer QEDs. In contrast, non-archers may not be able to inhibit these automatic error corrections, which are time consuming, lead to poorer prediction and, consequently, poorer, more variable performance. These distracting effects may be reflected by the presence of eye movements outside of the QE.

The fact that the QE is associated with the absence of eye movements (>1°) and is often referred to as a ‘fixation’ suggests that oculomotor control and, likely, attention control are involved in QE, in line with a plethora of published reports highlighting a central role between visuospatial attention and the generation of saccades (Deubel and Schneider 1996; Jonikaitis and Deubel 2011; Kowler et al. 1995; Rizzolatti et al. 1987). In addition, the inhibition of oculomotor responses plays an important role in maintaining focused attention to be able to make predictions and plan a motor response. Thus, in complex aiming tasks, skill-related effects are a result of the superior ability to override bottom-up attention capture via their top-down goal-directed attention control (dorsal and ventral attention control systems; Corbetta and Shulman 2002). The early QE onsets and longer QEDs of the expert group may have been implemented as a result of the perceived complexity of the task, as previously reported by Williams et al. (2002) and Klostermann et al. (2013), but may also point to a superior ability to inhibit other responses intruding into the motor programme. In contrast, the late QE onset in non-archers and in novices during HN compared to experts suggest that they needed extra time to position the joystick and achieve attentional focus, which may also be the case in the field. Therefore, in our tasks, the misalignment of attention (to central/critical cues as explained by Ryu et al. 2016) caused by online corrections and crosshair noise may be costly and result in larger performance errors.

Vickers (1992, 1996) supported this inhibition hypothesis after observations of a QE offset during movement initiation, that is, eye movements outside of the QE threshold were made once the motor response was executed. It follows that the maintenance of attention via stable gaze (QE) allows for a more efficient integration of online information into the motor programme, which may explain the archers’ superior performance. However, these fixation/inhibition abilities attributed to skilled individuals need to be further investigated. Previously, researchers investigating anxiety and gaze provide some evidence to suggest that inhibitory control is taking place during the QE, with performance improving after QE training under high anxiety conditions (see Vine et al. 2014). Anxiety has been associated with deficits in attention control and, in particular, inhibition (Berggren et al. 2013; Bishop 2009) due to competing cognitive resources. Additionally, greater cognitive loads have been shown to increase errors (inhibition) in antisaccade tasks (Berggren et al. 2013).

The beneficial effects of this “quiet” gaze on performance is evident in a number of QE training studies in which training consists of directing gaze to one location and maintaining that gaze for a longer period (Causer et al. 2011; Moore et al. 2012; Vine and Wilson 2011). This training is believed to maintain focused attention on critical cues and away from distractors. Similarly, a study implementing peripheral blurring of training videos has been shown to improve performance (decision making skills) in novice basketball players (Ryu et al. 2016). Ryu et al argued that this manipulation ensured the alignment of gaze and attention to critical central cues, overriding the conscious engagement with peripheral distractors. Further manipulations of target attention/inhibition mechanisms in QE across distinct skill levels may provide insight into the links between QE and attention, how these skills are acquired and used during such aiming tasks.

In line with previous notions that time spent preparing a movement facilitates the development of appropriate actions to minimize errors (Battaglia and Schrater 2007), and in line with the QE programming hypothesis (Williams et al. 2002), our results suggest that experts use aiming time more efficiently, compared to non-archers. We note, however, that the inhibition and programming hypotheses to explain the benefits of the QED on performance are not exclusive, but that attention/inhibitory mechanisms are needed for effective predictions and, thus, accurate programming. Additionally, it is likely that online control and monitoring are important mechanisms that can modulate QED. Further manipulations limiting feedback and/or using other kinds of sensory feedback (proprioceptive) could provide further insight into this notion. This interpretation suggests interactions between top-down and bottom-up control networks (or dorsal and ventral streams; Corbetta and Shulman 2002) during target selection and computations for movement parameterization during the QE in goal-directed actions. We have presented an experimental platform in which further examination of these issues can take place.

## Conclusions

Our findings support the growing literature based on the effectiveness and skill-related differences of longer QEDs in aiming tasks. We suggest that the QE is a gaze strategy that allows for the accurate programming and timely selection of a motor response using predictive online control. Suppression of intrusive responses during the preparation–selection period allows for this programming to take place, reflecting the timely inclusion of afferent signals used for prediction. The fact that we were able to see these QED differences in our field test and in a computer-based archery task now offers the potential to study in more detail the gaze strategies implemented by expert performers and the underlying mechanisms. Understanding how experts strategically control gaze and assign their cognitive resources could be exploited in many other domains outside of sport (e.g. arthroscopic surgery) and could lead to better focused training programmes.
